# Endovascular revascularization strategies using catheter-based thrombectomy versus conventional catheter-directed thrombolysis for acute limb ischemia

**DOI:** 10.1186/s12959-021-00349-9

**Published:** 2021-12-04

**Authors:** Maofeng Gong, Xu He, Boxiang Zhao, Jie Kong, Jianping Gu, Guoping Chen

**Affiliations:** Department of Interventional and Vascular Radiology, Nanjing First Hospital, Nanjing Medical University, Jiangsu 210006 Nanjing, People’s Republic of China

**Keywords:** Acute limb ischemia, Endovascular treatment, Catheter-based thrombectomy, Percutaneous mechanical thrombectomy, Catheter-directed thrombolysis

## Abstract

**Background:**

Acute limb ischemia (ALI) is an important clinical event threatening both life and the affected limbs, but the optimal treatment for ALI remains undefined. The aim of this study was to compare the safety and effectiveness of thrombectomy approaches via either catheter-based thrombectomy (CBT) or catheter-directed thrombolysis (CDT).

**Methods:**

A total of 98 patients (mean age 69.7 years, 60 male) who underwent endovascular intervention for ALI from January 2015 to July 2019 were included. Of these, 57 were treated with primary CBT via a large-bore catheter, an AngioJet catheter or Rotarex catheter, and/or underwent low-dose CDT, and 41 were treated with primary CDT. The safety and effectiveness of CBT compared to conventional CDT and other various endovascular techniques were evaluated.

**Results:**

More Rutherford IIb patients were treated with primary CBT (68.4%) than CDT (26.8%; *P* < .001). Patients who underwent primary CDT achieved a higher technical success rate than those who underwent primary CBT in a shorter procedure time (*P* < .001), whereas 42.1% of patients who underwent CBT did not need adjunctive CDT. The duration and dosage of adjunctive CDT in the CBT group were significantly decreased compared with those in the primary CDT group (both *P* < .001), and the CBT group achieved a shorter in-hospital length of stay (*P* < .001). Subgroup analysis revealed that patients treated with AngioJet and Rotarex catheters achieved slightly lower dosages, shorter CDT durations and shorter in-hospital stay lengths than those treated with large-bore catheters (*P* > .05). Clinical success was estimated to be achieved in 98.2% of patients who underwent CBT, which is similar to the 97.6% estimated in those who underwent primary CDT (*P* = 1.000), and this finding was similar among the CBT subgroups. Patients who underwent CBT had a higher procedure-related distal embolization rate and economic cost than those who underwent primary CDT (*P* < .05), but it had slightly fewer complications than those who underwent primary CDT (*P* = .059), especially minor complications (*P* = .036). The freedom from amputation at 6 and 12 months for CBT and CDT was assessed (93.0% vs 90.2% respectively, *P* = .625; 89.5% vs 82.9%, respectively, *P* = .34,). Comparable limb salvage was found for different techniques of large bore catheters, AngioJet catheters and Rotarex catheters. The Kaplan-Meier table analysis also showed similar limb salvage rates between groups.

**Conclusions:**

Endovascular treatment of ALI with the use of catheter-based therapies is an effective modality that can reduce the requirement for thrombolysis, with expected reductions in hemorrhagic complications, but at the risk of remediable distal emboli and increased economic cost. It has a similar clinical outcome to conventional CDT alone. Different CBT techniques have comparable efficacy but different adverse event profiles.

**Supplementary Information:**

The online version contains supplementary material available at 10.1186/s12959-021-00349-9.

## Article highlights

**Type of Research:** Single-center retrospective cohort study.

**Key findings:** CBT was successful as a stand-alone first-line endovascular technique in close to one-half of patients, with the remainder of patients requiring adjunctive CDT due to residual thrombus or emboli into distal small arteries where the CBT catheters could not safely reach. CBTs had the advantages of pronounced reduction of large volumes of thrombus in a moderate time and quicker return of blood flow and had comparable limb salvage rates, but at the risk of remediable distal emboli and increased economic cost. In contrast, primary CDT had greater technical success but was associated with more bleeding complications, including one bleeding-related death. Moreover, comparisons among CBT techniques revealed comparable outcomes in ALI patients regardless of which of the 3 modalities were used as first-line treatment but showed that the modalities had different adverse event profiles.

**Take home message:** Endovascular treatment of ALI with the use of catheter-based therapies is an effective modality that can reduce the requirement for thrombolysis, with expected reductions in hemorrhagic complications, but at the risk of remediable distal emboli and increased economic cost. It has a similar clinical outcome to conventional CDT alone. Regarding Rutherford IIb ischemia, CBT may have an advantage over CDT. In terms of CBT modalities, different techniques have comparable efficacy but have different adverse event profiles.

**Table of contents summary:** This retrospective single-center study analyzed the endovascular revascularization and outcomes of 98 acute limb ischemia patients. The study suggests that endovascular treatment of ALI with the use of catheter-based therapies is an effective modality that can reduce the requirement for thrombolysis, with expected reductions in hemorrhagic complications, but at the risk of remediable distal emboli and increased economic cost. It has a similar clinical outcome to conventional CDT alone. Regarding Rutherford IIb ischemia, CBT may have an advantage over CDT. In terms of CBT modalities, different techniques have comparable efficacy but have different adverse event profiles.

## Introduction

Acute limb ischemia (ALI), referred to as ischemia symptoms that emerge within 2 weeks, is one of the most common arterial emergencies [1, 2]. The most common causes of ALI are embolism, thrombosis of native arteries or reconstructions, peripheral arterial aneurysm, dissection and traumatic arterial injury [3]. ALI manifesting as “6P syndrome” can be catastrophic, with a potential threat to limb viability due to insufficient time for new vessel growth to compensate for the sudden interruption of limb perfusion [4, 5]. Ischemia is graded clinically according to the Rutherford ALI classification system. For acute, viable or marginally threatened ALI, timely recognition and revascularization aiming at restoring perfusion is recommended (Class I) [1, 2, 5].

ALI is a potential lethal event leading to not only amputation (12–50% of cases) but also death (20–40% of cases) without prompt treatments [4]. A majority of adverse outcomes occur within the initial days after presentation, indicating that intervention time is limited. Therefore rapid and effective revascularization following an episode of ALI is pivotal, as it will most likely improve the prognosis. However, determination of the optimal option for revascularization remains particularly challenging [1]. Surgical and endovascular approaches have been a longstanding topic of debate; both appear equally effective in the ALI patient population [6, 7] but have different adverse event profiles. Two recent guidelines, the European Society of Cardiology (ESC) and the European Society for Vascular Surgery (ESVS) guidelines [1, 8], have put a spotlight on ALI, and an evolution towards less invasive interventions has taken place, which may be an opportunity to encourage endovascular interventions in addition to surgery [9].

For endovascular approaches, conventional catheter-directed thrombolysis (CDT) is one of the most well established and employed techniques [10, 11]. Recently, a variety of new endovascular modalities aimed at mechanical disruption of the thrombus have emerged, and catheter-based thrombectomy (CBT) techniques, including thromboaspiration, microfragmentation, pharmacomechanical thrombectomy and ultrasound-accelerated CDT, have emerged and been made more available [12]. Even if all of the above techniques have been widely applied in clinical practice, relatively little is known about critical points such as safety and competitive device performance. Thus, the primary objective of this study was to compare the safety and effectiveness of CBT to conventional CDT in the management of ALI, as well as to evaluate various endovascular techniques with large bore catheters, Rotarex catheters and AngioJet catheters.

## Methods

### Patients and study design

**Fig. 1:**
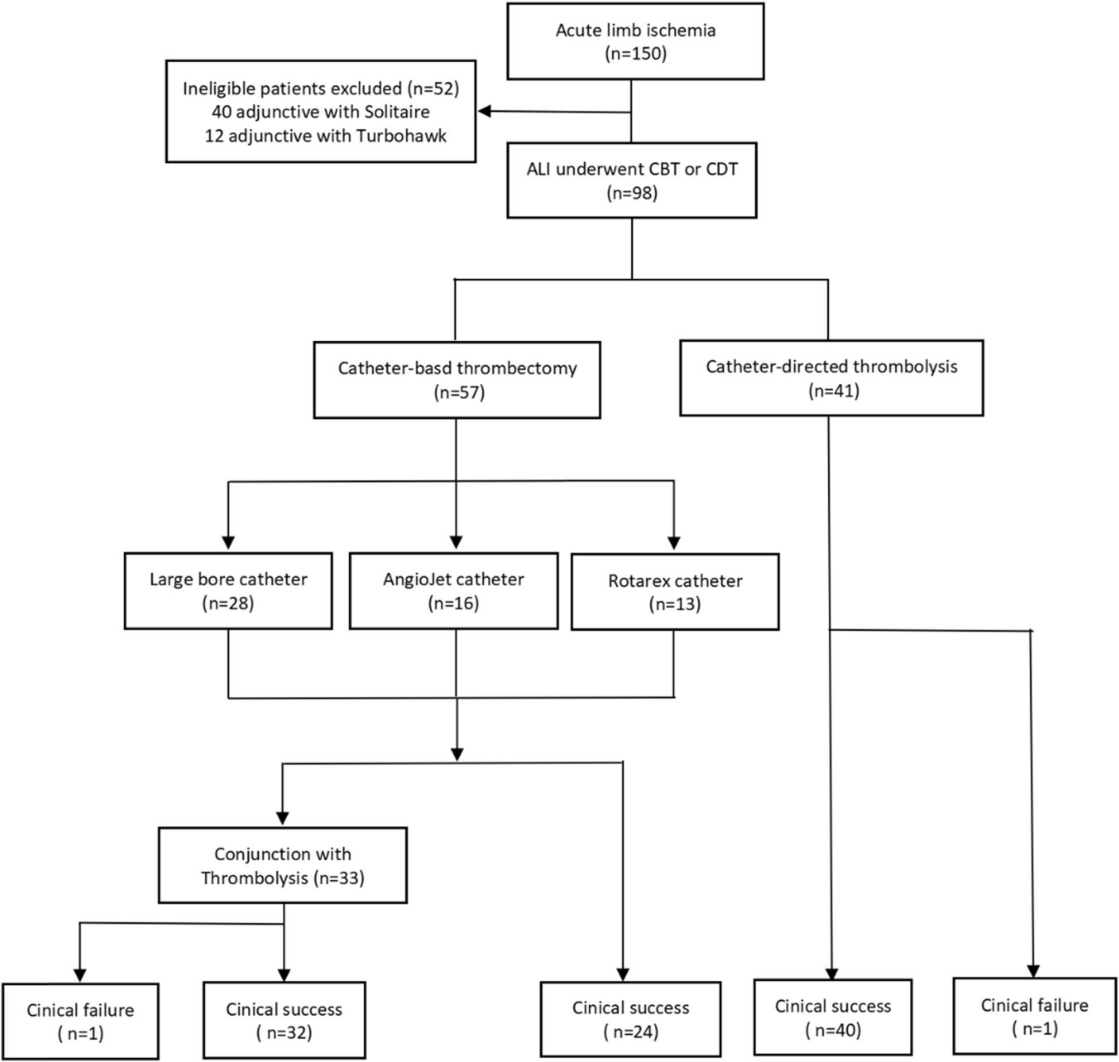
Study Flowchart. CBT = catheter-based thrombectomy; CDT = catheter-directed thrombolysis

This was a retrospective cohort study which included confirmed ALI patients who underwent endovascular revascularization as first-line treatment at a single academic center from January 2015 to July 2019. All patients underwent treatment via the CBT and/or CDT approach, and data were retrospectively derived from the medical database system and paper records. This data collection protocol was approved by the institutional review board, and the need for informed consent was waived owing to the retrospective nature of this study. A study flow-chart is shown in Fig. 1.

### Management strategies details

ALI was initially assessed in the majority of patients with urgent duplex ultrasound and CT arteriography (CTA). Then, the therapeutic approach was left to the discretion of the treatment group, consisting of 3 interventional radiologists with at least 15 years of experience. The decision to proceed with either CBT or CDT as the first-line treatment was determined by the interventional radiologist operators based mainly on the degree of ischemia, experience and devices available. Although the procedural details varied slightly in different cases depending on individuality therapy, procedures were largely similar, and the exact CBT reperfusion details are shown in Supplementary Table 1. If residual in situ thrombus or distal embolization was found after embolectomy, further widely used alternative conjunction with CDT was considered.

Patients who underwent conventional CDT as first-line treatment were infused a bolus dose of 5 mg of recombinant tissue-type plasminogen activator (rt-PA) (Actilyse; Boehringer Ingelheim; Ingelheim, Germany) via a multiple-side hole thrombolytic catheter (Uni*Fuse; Angio-Dynamic; Latham, NY). Both CDT techniques subsequently received a continuous infusion of reduced-dose rt-PA for further treatment. Thrombolytic therapy with rt-PA was administered as follows: 20 mg of actilyse in 500 ml of 0.9% saline was administered through the reserved thrombolysis catheter at an infusion rate of 0.01 mg/kg/h; the maximum rate was no more than 1.0 mg/h. Thrombolysis with actilyse was only administered when the fibrinogen level, which was monitored at 12–24 hourly intervals, was greater than 1.0 g/L. Follow-up angiography was performed to assess efficacy every 24 h. In the absence of any contraindications, CDT was continued if the thrombus load remained and discontinued when treatment was complete or in the event of an adverse event.

If more than the indicated minimum of 50% stenosis remained after thrombus removal, balloon angioplasty and/or stenting was performed. During each thrombolysis therapy or at the end of CDT, low molecular weight heparin (Hebei Changshan Biochemical Pharmaceutical; Shijiazhuang, China) therapy at a dose of 100 IU/kg per 12 h was started immediately. In the absence of local hemorrhagic complications, dual antiplatelet therapy (aspirin 100 mg/d and clopidogrel 75 mg/d) and statin therapy (atorvastatin, 20 mg/d) were prescribed after discharge. Outpatient follow-ups were conducted by our center at 1, 3, 6, and 12 months postprocedure via ankle-brachial index (ABI), duplex ultrasound and/or CTA; additional procedures and complications were recorded during follow-up.

### Definitions of outcome and safety measures

The clinical severity of ALI was classified by the Rutherford classification system details are listed in Supplementary Table 2 [5]. Technical success of the primary procedure was defined as complete in situ thrombus clearance of CBT or CDT alone, and adjunctive treatment of CDT to achieve thrombus clearance for CBT was considered to indicate assistant technical success. Clinical success (improvement in clinical status) was defined as the absence and/or relief of symptoms related to ALI according to accepted guidelines [5, 11], or an improvement in the Rutherford grade of at least one category with objective evidence of hemodynamic change (at least 0.1 increase in ABI). Limb salvage was defined as freedom from major amputation (performed above the ankle), and maintained functional autonomy (walking or standing) was determined in accordance to accepted guidelines [5]. The need for necessary additional techniques (such as balloon angioplasty and/or stenting) to treat underlying chronic disease to obtain sufficient distal perfusion within the same hospital stay was recorded but not considered clinical failure. Safety was classified as major or minor complications in accordance with the criteria of the Society of Interventional Radiology (SIR) classification scale [13].

### Statistical analysis

The *SPSS* statistical software package (version 23.0; *SPSS* statistical software, Chicago, Illinois, USA) was used to perform all statistical analyses in this study. Continuous variables are expressed as the mean ± standard deviation. Qualitative variables are presented as numbers and percentages. When assessing the correlation between pre- and postprocedural variables, a paired *t-*test was used. The significance of qualitative variables was tested with a *chi-square* test or *Fisher’s* exact test. Findings with a *P* value less than 0.05 were deemed statistically significant.

## Results

### Baseline characteristics and ischemia classification of patients

A total of 98 patients [mean age 69.7 years, 61.2% male (*n* = 60)] with ALI who underwent endovascular revascularization with either CBT or CDT were included. Of those patients who underwent CBT as first-line treatment, 28 were treated with large-bore catheters, 16 with AngioJet catheters, and 13 with Rotarex catheters, and 41 underwent conventional CDT solely as the first-line treatment. The demographics, comorbidities, presentation and lesion characteristics of 98 patients are summarized in Table 1. Procedure characteristics and outcomes are outlined in Tables 2, and 3 summarizes outcomes by subgroups of interest.

**Table 1:** Demographics, Comorbidities, Presentation and Lesion Characteristics of ALI Patients

Characteristic	Primary CBT (*n* = 57)	Primary CDT (*n* = 41)	*p* value
Age, y, mean ± SD (range)	68.89 ± 11.88	70.63 ± 10.87	.461
Male sex	35 (61.4%)	25 (61.0%)	.966
Duration of symptoms at presentation, h, mean ± SD (range)	37.2 ± 32.8	31.0 ± 28.2	.332
Risk factors			
Diabetes mellitus	20 (35.1)	12 (29.3)	.545
Coronary artery disease	19 (33.3)	13 (31.7)	.886
Rheumatic heart disease	9 (15.8)	5 (12.2)	.616
Previous cerebrovascular accident	7 (12.3)	3 (7.3)	.644
Renal insufficiency	5 (8.8)	3 (7.3)	1.00
Hypertension	38 (66.7)	31 (75.6)	.339
Hyperlipidemia	18 (31.6)	13 (31.7)	.989
Diagnosed atrial fibrillation	35 (61.4)	27 (65.9)	.652
Current diagnosis of cancer	3 (5.3)	1 (2.4)	.858
History of smoking	17 (29.8)	13 (31.7)	.842
History of peripheral artery disease	20 (35.1)	12 (29.3)	.545
Thrombosed segment			
Iliac	7 (12.3)	3(7.3)	.644
Iliofemoral (including iliofemoral stents)	42 (73.7)^a^	24 (58.5)	.115
Femoropopliteal	8 (14.0)	12 (29.3)	.065
Crural and Tibial	0 (0)	2 (4.9)	.173**
Ischemia level (Rutherford category)			
I (viable limb)	1 (1.8)	4 (9.8)	.076
II a (marginally threatened limb)	17 (29.8)	26 (63.4)	.001*
II b (immediately threatened limb)	39 (68.4)	11 (26.8)	.000*

*ALI* Acute limb ischemia, *CBT* Catheter-based thrombectomy, *CDT* Catheter-directed thrombolysis

Continuous data are presented as the means ± standard deviations; categorical data are given as the counts (percentage)

*P < .05; ** Fisher exact

^a^Includes two patients of thrombus in stents

**Table 2:** Procedure Characteristics by Treatment Approach and Outcomes

Characteristics	Primary CBT (*n* = 57)	Primary CDT (*n* = 41)	*p* value
Technical success of thrombus/embolus removal			
With primary intervention only	24 (42.1)	39 (95.1)	.000
Including adjuvant thrombolysis	57 (100)	41 (100)	1.000
Adjunctive angioplasty/stenting after thrombus/embolus removal	47 (82.5)	33 (80.5)	.804
Duration of operation procedure, h	1.89 ± .52	1.32 ± .44	.000
Total duration of thrombolysis, d	1.74 ± .98	3.07 ± 1.38	.000
Rt-PA dose, mg	14.14 ± 5.75	29.27 ± 11.70	.000
ABI scores			
Pretreatment	.29 ± .09	.31 ± .08	.747
Treatment completion	.72 ± .16	.66 ± .13	.101
Clinical success	56 (98.2)	40 (97.6)	1.000
Limb salvage at 6 months	53 (93.0)	37(90.2)	.625
Limb salvage at 12 months	51 (89.5)	34 (82.9)	.346
30-day complications	10 (17.5)	14 (34.1)	.059
Minor (SIR A, B: nominal or no therapy, no consequence)	6 (10.5)	11(26.8)	.036
Major (SIR C, D, E: requires therapy or permanent sequelae)	3 (5.3)	2 (4.9)	1.000
Major-death (SIR F: death)	1 (1.8)	1 (2.4)	1.000
Procedure- related distal embolization	14 (24.6)	2 (4.9)	.009
In-hospital length of stay, d	4.97 ± .13	6.04 ± .95	.000

*CBT* Catheter-based thrombectomy, *CDT* Catheter-directed thrombolysis

Continuous data are presented as the means ± standard deviations; categorical data are given as the counts (percentages)

**Table 3:** Comparisons of Outcomes by Treatment Approach (large bore catheter and Rotarex and AngioJet devices)

Characteristic	Large bore catheter	Rotarex catheter	AngioJet catheter	*p* value
Age, y, mean ± SD (range)	69.7 ± 13.2	67.6 ± 9.9	68.8 ± 11.7	.862
Sex, male	17 (60.7)	9 (56.3)	9 (69.2)	.768
Duration of symptoms at presentation, h, mean ± SD (range)	31.2 ± 36.5	50.8 ± 30.0	36.7 ± 25.8	.208
Ischemia level (Rutherford category)				
I (viable limb)	1 (3.6)	0 (0)	0 (0)	.590
II a (marginally threatened limb)	11 (39.3)	3 (18.8)	3 (23.1)	.292
II b (immediately threatened limb)	16 (57.1)	13 (81.3)	10 (76.9)	.186
Procedure length (h, mean ± SD)	1.74 ± .54	2.03 ± .53	2.04 ± .38	.103
Technical and clinical success with initial endovascular procedure only	13 (46.4)	5 (31.3)	6 (46.2)	.584
Total duration of thrombolysis, d	.775 ± .20	.56 ± .16	.61 ± .22	.092
rt-PA dosage, mg	16.67 ± 5.56	12.31 ± 5.63	12.1 ± 4.88	.076
In-hospital length of stay, d	5.21 ± .92	4.78 ± .71	4.69 ± 1.18	.165
ABI scores				
Pre procedure	.30 ± .11	.29 ± .09	.28 ± .04	.743
Treatment completion	.68 ± .15	.75 ± .17	.75 ± .16	.179
Limb salvage at 6 months	26 (92.9)	15 (93.8)	12 (92.3)	.988
Limb salvage at 12 months	25 (89.3)	14 (87.5)	12 (92.3)	.915
Procedure-related complications				
Minor (SIR A, B: nominal or no therapy, no consequence)	3 (10.7)	2 (12.5)	1 (7.7)	.912
Major (SIR C, D, E: requires therapy or permanent sequelae)	1 (3.6)	1(6.3)	1 (7.7)	.841
Major-death (SIR F: death)	1 (3.6)	0 (0)	0 (0)	–
Procedure- related distal embolization	6 (21.4)	5 (31.3)	3 (23.1)	.765

Data are presented as the mean ± standard deviation or number (percentage)

The mean duration of ischemia symptoms before presentation was slightly longer in the CBT group of than that in the CDT group (37.2 ± 32.8 h vs 31.0 ± 28.2 h, respectively; *P* = .332). Within the CBT group, the Rotarex subgroup had a longer duration of ischemia (50.8 ± 30.0 h, *P* = .208). With regard to etiology, the major risk factors for the development of ALI were hypertension (70.4%), diagnosed atrial fibrillation (63.3%) and coronary artery disease (32.7%). In the majority of cases, the thrombosed segment primarily resided in the iliac, iliofemoral arteries for the CBT group, and iliofemoral or femoropopliteal arteries for the CDT group; two cases in the CBT group had iliofemoral thrombus of existing stents, and two cases in the CDT group had distal artery thrombus. There were no statistically significant differences in baseline characteristics between the two groups or among the subgroups of large-bore catheters, AngioJet catheters and Rotarex catheters (*P* > .05). With regard to the ischemia classification of patients, the primary CDT group had a slightly higher proportion of patients with Rutherford grade I ischemia (*P* = .076); there were fewer Rutherford grade IIa patients treated with primary CBT (*n* = 17, 29.8%) than those treated with CDT (*n* = 26, 63.4%; *P* = .001), and more Rutherford grade IIb patients were treated with the use of primary CBT (*n* = 39, 68.4%) than those treated with CDT (*n* = 11, 26.8%; *P* < .001).

### Outcomes of technical and clinical success

Procedure characteristics and outcomes are outlined in Tables 2 and 3. Patients who underwent primary CDT achieved a higher technical success rate (*n* = 39, 95.1%) than those who underwent primary CBT (*n* = 24, 42.1%) in a shorter time [1.32 ± .44 h versus 1.89 ± .52 h; *P* < .001)]. In the subgroup analysis, large-bore catheters had slightly shorter procedure lengths than AngioJet or Rotarex catheters. In the 57 patients who underwent primary CBT and experienced technical failure, 33 patients required adjunctive CDT due to either residual in situ thrombus (*n* = 19) or dislodged distal thrombus (*n* = 14). At completion, additional balloon angioplasty and/or secondary stenting for underlying chronic disease to obtain sufficient distal reperfusion was performed in 81.6% of patients (*n* = 80), without a significant difference between the two groups (*P* = .804).

The duration and dosage of adjunctive CDT were significantly lower when compared to those for primary CDT (duration was 1.74 ± .98 days vs 3.07 ± 1.38 days, mean dosage rt-PA 14.14 ± 5.75 mg vs 29.27 ± 11.70 mg, both *P* < .001). The CBT group also demonstrated a shorter in-hospital length of stay (*P* < .001). In the subgroup analysis, AngioJet and Rotarex catheters achieved slightly lower dosages, shorter CDT durations and shorter length of in-hospital stay than large-bore catheters. Clinical success estimates were achieved in 98.2% of patients (*n* = 56) who underwent primary CBT, which was similar to the 97.6% (*n* = 40) success in those who underwent primary CDT (*P* = 1.000). These were similar among the subgroups of CBT, despite differences in the proportions of patients with Rutherford IIa and Rutherford IIb ischemia treated with each modality. Of these, the clinical success rates of patients in Rutherford IIa and IIb were 100% and 97.4%, respectively (*P* = 1.000). In both groups, ABI was significantly improved from preprocedure measurements to those after treatment completion (*P* < .001); however, the difference was not statistically significant between each other or among the subgroups (*P* > .05).

The average procedural costs including devices and thrombolytic agents and total cost of hospitalization were CNY ¥ 46,522.1 ± 14,724.8 and CNY ¥ 69,633.4 ± 27,460.6 per patient for patients who underwent primary CBT, which were higher than CNY ¥ 35,249.9 ± 8177.2 and CNY ¥ 49,734.3 ± 10,710.8 in those who underwent primary CDT (*P* < .05).

### Complications and treatment therapy

The 30-day complications recorded are listed in Tables 2 and 3. Patients who underwent primary CBT had slightly fewer complications than those who underwent primary CDT (*P* = .059), especially for minor complications (*P* = .036). Minor complications for the CBT group included vessel spasm (*n* = 3), dissection of the superficial femoral artery (*n* = 1), hematuria (*n* = 1) and hematoma of the puncture site (*n* = 1); the rate of complications was significantly lower than that for CDT. Minor complications in the CDT group were presented mainly as hemorrhage events, including hematoma of the puncture site (diameter 3–5 cm, *n* = 6), pseudoaneurysm (*n* = 2), hematuria (*n* = 2) and calf hematoma (*n* = 1). Local pressure dressing was applied for observation and treatment of minor hemorrhage, and minor complications experienced less serious adverse consequences. The major complication except for distal embolization of the CBT group was compartment syndrome (*n* = 3) requiring surgery for calf fasciotomy. Two patients in the CDT group required transfusion of two units of red blood cell suspension due to major gastrointestinal hemorrhage. Fortunately, all these events resolved without permanent sequelae. One death in each group was recorded: one subject in the CBT group died from cardiac failure after amputation, which was unrelated to the procedure, and the one subject in the CDT group died from intracranial hemorrhage after CDT despite surgical decompression. CBT had a higher procedure-related distal embolization rate than those who underwent primary CDT (*P* = .009), in the subgroup analysis, Rotarex catheters had more procedure-related distal emboli than ﻿large-bore catheters and AngioJet catheters, yet the difference was not statistically significant among the 3 groups (*P* = .765). Owing to the small size of these vessels and underlying chronic disease, adjunctive CDTs were performed, the dislodged thrombus was well treated without permanent sequelae.

### Follow-up and limb freedom from amputation

**Fig. 2:**
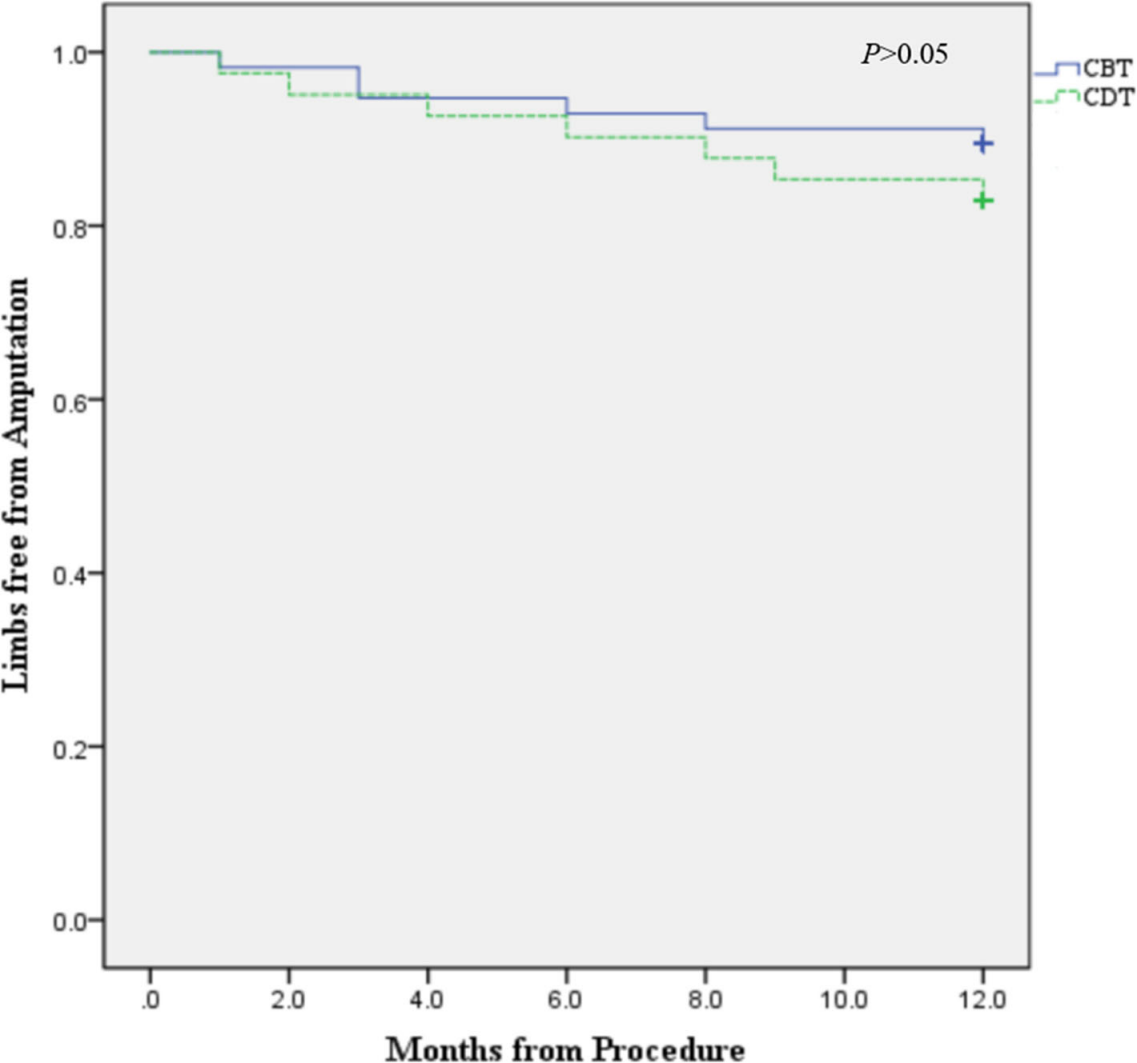
Kaplan-Meier estimates of limbs salvage for the CBT and CDT cohort at 12 months. CBT = catheter-based thrombectomy; CDT = catheter-directed thrombolysis

No patients were lost to follow-up, and 98.0% (96/98) of patients were alive when discharged from the hospital. The reintervention rates at 6 months and 12 months were 9.2% (*n* = 9) and 17.3% (*n* = 17), respectively. A total of four patients in each group suffered major amputations at 6 months for the CBT and CDT groups and six and seven at 12 months, which were primarily attribute to intractable infection caused by diabetes, osteomyelitis or repeat embolism event. The freedom from amputation at 6 months in the CBT and CDT groups was 93.0 and 90.2%, respectively, and at 12 months was 89.5 and 82.9%, respectively (*P* > .05). The Kaplan-Meier analysis also showed similar limb salvage rates between groups (Fig. 2). Similarly, no significant differences were seen among groups *(P > .*05).

## Discussion

Present study demonstrated that CBT is successful as a stand-alone first-line endovascular technique in close to one-half of patients with ALI, with the remainder requiring adjunctive CDT due to residual thrombus or emboli into distal small arteries where the CBT catheters could not safely reach. CBTs had the advantages of pronounced reduction of large volumes of thrombus in a moderate time, speedy recanalization of blood flow and comparable limb salvage, but at the risk of remediable distal emboli and increased economic cost. In contrast, primary CDT had greater technical success but with more bleeding complications (statistically significant for minor bleeds), including one bleeding-related death. Moreover, the comparison among CBTs revealed comparable outcomes in ALI patients regardless of which of the 3 modalities were used as first-line treatment, but had different adverse event profiles. Because ALI might threaten limbs and life, especially quality of life, shared decision making between clinicians, patients and families based on the best available option for ALI patients seems to be important.

The strategy options for ALI treatment in our study depended mainly on the severity of ischemia. CDT for revascularization often takes time, and ischemia may progress during treatment if the thrombus is not removed in a timely manner [1, 2, 6]. In this study, 73.2% of CDT therapy was applied in patients with Rutherford grade I and IIa ischemia considering its inherent defects of slow opening of the lumen. In 68.4% of patients with Rutherford grade IIb, more emergency revascularization techniques, including large-bore catheters, Rotarex catheters and AngioJet catheters, were used as the preferred first-line methods to restore perfusion. Although clinical success and freedom from amputation in the Grip et al. [7] study were inferior in patients with Rutherford grade IIb than IIa ischemia, the present study demonstrates that outcomes were no worse for patients with Rutherford grade IIb ischemia. CBT was empirically performed for iliac and iliofemoral thrombosed segment and CDT was used primarily for iliofemoral and femoropopliteal segment. Patients who underwent CBT achieved similar outcomes, removing emboli/thrombus and opening the lumen with shorter procedure times than CDT. The strategy of CBT for Rutherford grade IIb ischemia seems to be a suitable alternative when initiated promptly.

In the present cohort study of patients treated by CDT and CBT, both techniques were shown to be useful. We compared CBT and CDT as the primary endovascular procedures, and the results indicated that technical success rates of CBT were 42.1%, which was lower than that of CDT as 57.9% of CBT patients underwent conjunctive CDT. This number was lower than that reported by Zehnder et al. [14], which may be attributed to a more stringent definition (not including adjunctive CDT) of technical success in the present study. A matched analysis comparing clinical success and limb salvage between the two groups showed slightly better outcomes and comparable limb salvage in the CBT group. The patients who were free from amputation at 6 months and 12 months was approximately 93.0% versus 90.2% and 89.5% versus 82.9% in both groups, which was similar to those of other published studies [15, 16]. While more patients with Rutherford grade IIb ischemia were considered, CBT attained a better outcome. These results indicated that it might be better to perform CBT initially than CDT. A limitation of CBTs was the inability to use the devices in the small-caliber arteries of the lower limbs; however, it was managed by conjunctive low-dose CDT. Even if adjuvant therapy was possible, and the procedure time tended to be shorter in CDT, CBT seemed to have the advantages of a lower CDT duration and rt-PA dosages than conventional CDT. Several studies have examined whether the risk factors for bleeding risk during CDT are related to the duration and dosages of CDT used [17, 18]. Approximately one-half of patients with ALI did not require adjunctive CDT, and the present study supported the major potential advantage of CBT, which, if successful as a stand-alone treatment, obviates the requirement for CDT with a concomitant reduction in the risk of hemorrhagic complications.

One study that compared CDT with or without pharmacomechanical thrombolysis using the AngioJet device showed that CBT increased technical success rates but at the cost of more distal emboli, causing embolization of both large and small particles. Notably, patients treated with CBT in our study also encountered distal emboli events, similar to a published study [16]. The dislodged thrombus was successful treated by adjunctive CDT, without permanent sequelae, which seems remediable. It should be noted that a variety of distal embolic protection devices have been developed for carotid artery stenosis and deep vein thrombosis, a situation of minor embolization and less serious consequences [19]. The use of distal embolic protection devices has been considered suitable but not yet advocated in ALI treatments. The mean rt-PA dosage was lower in CBT than in CDT. Although there was no significant difference in the frequency of major complications between the two groups, two cases of major hemorrhage and one death complication were noted in CDT, while no such cases were recorded in CBT. Furthermore, three patients who underwent CBT required calf compartment decompression due to compartment syndrome after reperfusion, which was not recorded in CDT. A possible explanation for this may lie in the differences in ischemia grade at presentation for each group; more patients with Rutherford grade IIb ischemia were present in the primary CBT group, and more ALI patients with Rutherford IIa ischemia were present in the primary CDT group. In addition to the severity of ischemia, another possible explanation may be related to the shorter time taken to achieve reperfusion with CBT compared with CDT, which may result in massive blood perfusion and exudation [20, 21]. The risks of CBT may lead to hyperkalemia, myoglobinuria, and renal damage [15], but these risks were not recorded in the present study.

Subgroup analysis evaluated within distinct patients showed that three techniques exhibited comparable outcomes but had different adverse event profiles. The PEARL registry study showed AngioJet catheter as a modality for treating ALI, with a technical success rate of 52%, and adjuvant CDT improved this success rate to 83% [15]. Rotarex devices were described with a technical success rate of 68.7% as a stand-alone technique but with additional thrombolysis in 90.5% [22]. Patients with ALI assigned to an initial large-bore catheter and AngioJet catheter tended to have improved technique success rates in the present study and had less need for adjunctive intervention. The Rotarex device revealed a lower technical success rate and a greater need for additional CDT due to distal artery emboli. A physiological circulation model study [23] revealed that the Rotarex system had slight advantages, but significantly more thromboemboli and vascular injuries; however, the AngioJet was more tissue preserving. Similar to our subgroup analysis, AngioJet and large bore catheter had slightly first pass recanalization and lower distal emboli when compared to Rotarex catheter in a “real-world” contemporary clinical setting.

Some limitations of the present study should be mentioned. Due to the aim of present study was to investigate the endovascular revascularization strategies, patients who underwent surgical revascularization were not included. The strategy employed mainly depended on the severity of ischemia and the expertise and facilities of the treating team, and the study was not randomized, which could have resulted in potential selection biases and confounding variables. Although the outcome in the subgroup analysis of PAT with a large-bore catheter and PMT with an AngioJet/Rotarex catheter was conducted, the conclusion was limited to a small subgroup of cases, which may need to be confirmed, and there is a need for future research in this field. Meanwhile, devices such as Penumbra/Indigo had not been utilized in the present study. Therefore, the thromboaspiration device used in the present study was limited to a simple large-bore catheter. Nevertheless, this study hopes to help clinicians choose between approaches for individual patients, but is inevitably hampered by a lack of robust data. The conclusions of the present study are limited due to the small, retrospective and nonrandomized analysis from a single center. These data may help prompt the design of RCTs that may differ from the guidelines.

## Conclusion

This study demonstrates that endovascular treatment of ALI with the use of catheter-based therapies is an effective modality that can reduce the requirement for thrombolysis, with expected reductions in hemorrhagic complications, but at the risk of remediable distal emboli and increased economic cost. It has a similar clinical outcome to conventional CDT alone, CBT was empirically performed for iliac and iliofemoral thrombosed segment and CDT was used primarily for iliofemoral and femoropopliteal segment. Regarding Rutherford IIb ischemia, CBT may have an advantage over CDT. In terms of CBT modalities, different techniques have comparable efficacy, but have different adverse event profiles. Insufficient data are available to determine a preference for a specific technique among CBTs. Future trials regarding ALI need to be designed carefully, ensuring comparable study groups, and should follow standardized practices of outcome reporting.

## Supplementary Information

**Additional file 1 Supplementary Table 1.** Techniques and devices for catheter-based therapy and catheter-directed thrombolysis of Acute Ischemia Patients. **Supplementary Table 2.** Rutherford categories of Acute Ischemia Patients.

## Data Availability

The datasets generated and analyzed during the current study are not publicly available, as the experimental data are related to other experiments that are progressing but are available from the corresponding author upon reasonable request.

## Abbreviations

ABI: Ankle-brachial index
ALI: Acute limb ischemia
ESC: European Society of Cardiology
ESVS: European Society for Vascular Surgery
LMWH: Low molecular weight heparin
MALE: Major adverse limb events
PTA: Percutaneous transluminal angioplasty
PAT: Percutaneous aspiration thrombectomy
RCT: Randomized controlled trial
rt-PA: Recombinant tissue plasminogen activator
SFA: Superficial femoral artery
