# Supplementary material for: Endovascular revascularization strategies using catheter-based thrombectomy versus conventional catheter-directed thrombolysis for acute limb ischemia
Source: Thromb J. 2021 Dec 4;19:96. doi: 10.1186/s12959-021-00349-9 (PMC8645071; doi:10.1186/s12959-021-00349-9)
Supplement: Supplementary file 1 — Additional file 1 Supplementary Table 1. Techniques and devices for catheter-based therapy and catheter-directed thrombolysis of Acute Ischemia Patients. Supplementary Table 2. Rutherford categories of Acute Ischemia Patients. [file 12959_2021_349_MOESM1_ESM.docx]

| **Supplementary Table 1.** Techniques and devices for catheter-based therapy and catheter-directed thrombolysis of Acute Ischemia Patients. | | | |
| --- | --- | --- | --- |
| Technique (Type) | Device used | Angiographic thrombus characteristics | Procedural details |
| Catheter-directed thrombolysis (CDT) | Uni*fuse catheter (4F);  Fountain catheter (4F) | Slight, fresh thrombus/emboli | The distal end of the guidewire was placed over the site of the thrombus, a multiple side hole infusion catheter was delivered traversing the occlusion and low-dose alteplase was delivered via the catheter. |
| Thrombo-aspiration thrombectomy (CBT) | Guiding catheter (8F), syringe (50 mL) | Massive, fresh thrombus/emboli | An 8F guiding catheter combined with a regular 50 mL syringe with a detachable hemostatic valve for suction is used to slowly withdraw the clot through the introducer sheath to aspirate it from the vessel. |
| Microfragmentation thrombectomy (CBT) | Rotarex catheter (8F) (Straub Medical, Switzerland) | Massive, slight older thrombus/emboli | Rotarex catheter was introduced over a 0.018-inch guidewire and then advanced across the thrombus segment. The catheter was gently withdrawn and pulled back during aspiration. |
| Pharmaco-mechanical thrombectomy (CBT) | AngioJet (6F) thrombectomy with power pulse thrombolysis (Boston Scientific, Fremont, CA) | Massive, fresh thrombus/emboli | Care was taken to advance the AngioJet catheter slowly through the thrombosed segment (only in vessels estimated to be greater than 6 mm in diameter). Then, 25 million units of urokinase was delivered under the power pulse model and waiting 30 minutes. The pump unit was active during slow catheter passages (3 mm/s to 5 mm/s) across the thrombus segment in a distal-to-proximal or adverse direction and under fluoroscopic guidance. Individual run times were monitored and kept at less than 20 s. |
| Combined techniques | CBT combined with reduced-dose thrombolysis | Situ thrombus residual or distal embolism | Alteplase 20 mg diluted in 500 ml of 0.9% saline was administered through catheter at an infusion rate of 0.01 mg/kg.h following CDT; the maximum rate was no more than 1.0 mg/h. |

CDT = catheter-directed thrombolysis, F = French (refers to catheter diameter).

This is an identical replica of the table in the 1997 publication by Rutherford et al.,^2^ with the exception of the asterisks (*).

| **Supplementary Table 2.** Rutherford categories of Acute Ischemia Patients. | | | | | |
| --- | --- | --- | --- | --- | --- |
| Category | Description/prognosis | Findings | | Doppler signal | |
|  |  | Sensory loss | Muscle weakness | Arterial | Venous |
| I. Viable | Not immediately threatened | None | None | Audible | Audible |
| II. Threatened |  |  |  |  |  |
| a. Marginally | Salvageable if promptly treated | Minimal (toes) or none | None | Inaudible | Audible |
| b. Immediately | Salvageable with immediate revascularization | More than toes, associated with rest pain | Mild, moderate | Inaudible | Audible |
| III. Irreversible | Major tissue loss or permanent nerve damage inevitable | Profound, anesthetic | Profound, paralysis (rigor) | Inaudible | Inaudible |
